# Effects of Corchorusoside C on NF-κB and PARP-1 Molecular Targets and Toxicity Profile in Zebrafish

**DOI:** 10.3390/ijms232314546

**Published:** 2022-11-22

**Authors:** Nathan P. Mirtallo Ezzone, Gerardo D. Anaya-Eugenio, Ermias Mekuria Addo, Yulin Ren, A. Douglas Kinghorn, Esperanza J. Carcache de Blanco

**Affiliations:** Division of Medicinal Chemistry and Pharmacognosy, College of Pharmacy, The Ohio State University, Columbus, OH 43210, USA

**Keywords:** corchorusoside C, zebrafish, NF-κB, PARP-1, caspase-1

## Abstract

The present study aims to continue the study of corchorusoside C (**1**), a cardenolide isolated from *Streptocaulon juventas*, as a potential anticancer agent. A mechanistic study was pursued in a zebrafish model and in DU-145 prostate cancer cells to investigate the selectivity of **1** towards NF-κB and PARP-1 pathway elements. Compound **1** was found to inhibit the expression of IKKα and NF-κB p65 in TNF-α induced zebrafish and inhibit the expression of NIK in vitro. The protein expression levels of XRCC-1 were increased and p53 decreased in DU-145 cells. XIAP protein expression was initially decreased after treatment with **1**, followed by an increase in expression at doses higher than the IC_50_ value. The activity of caspase-1 and the protein expression levels of IL-18 were both decreased following treatment of **1**. The binding interactions for **1** to NIK, XRCC-1, p53, XIAP, and caspase-1 proteins were explored in molecular docking studies. Additionally, the toxicity profile of **1** in zebrafish was favorable in comparison to its analog digoxin and other anticancer drugs at the same MTD in zebrafish. Overall, **1** targets the noncanconical NF-κB pathway in vivo and in vitro, and is well tolerated in zebrafish supporting its potential in the treatment of prostate cancer.

## 1. Introduction

Cancer is a complex set of diseases characterized by the proliferation of abnormal cells in the body [1]. Numerous risk factors influence the ability of cancer cells to proliferate, including the use of tobacco, excess body weight, and inherited genetic mutations [1]. Cancer is one of the most common causes of death in the United States, second to only cardiovascular disease [1]. In 2022 alone, the United States is expected to experience over 1.9 million new cases of cancer and 609,360 cancer-related deaths [1]. Prostate cancer is expected to account for nearly 27% of the new cases of cancer in men in 2022, and resultantly, is projected to be the second leading cause of cancer-related death in men [1]. Thus, there is a continued need for safe and effective prostate cancer therapies.

Natural products throughout history have been a vital source of novel compounds used for the treatment of cancer [2]. In the timeframe from 1946 to September of 2019, 321 anticancer drugs have been approved worldwide, of which 206 (approximately 64.2%) can be attributed to naturally inspired sources, whether the drug is an unaltered natural product, a natural product derivative, a synthetic drug with a natural product pharmacophore, or a mimic of a natural product [3]. Common examples of plant-derived anticancer natural product compounds include paclitaxel from the pacific yew *Taxus brevifolia* Nutt., and vinblastine and vincristine from *Vinca rosea* L. [2].

Recently, cardiac glycosides, chemically classified as cardenolides, have been evidenced to have a role in the prevention and/or treatment of cancer [4,5]. For example, a retrospective study involving 47,884 men was performed to examine the risk of prostate cancer development and prostate cancer-related death in patients administered digoxin from foxglove (*Digitalis lanata* L.), for its conventional use as an antiarrhythmic agent [6]. The results of the study suggested regular users of digoxin experienced a relatively lower risk of developing prostate cancer compared to nonusers [7]. Moreover, the cardiac glycosides bufalin, digitoxin, cinobufagin, oleandrin, ouabain, and digoxin have been shown to have antiproliferative activity in vitro on androgen-dependent and -independent prostate cancer cell lines [5]. Also, cardenolides, as a class of natural products, are known for their role in the treatment of heart failure and hypertension due to their role in increasing cardiac output [8]. Cardenolides have been shown to inhibit Na^+^/K^+^-ATPase activity, resultantly stimulating an increase in intracellular Na^+^, followed by an increase in intracellular Ca^2+^, and a positive inotropic activity on cardiac muscle [8]. Throughout the 20th century, digoxin was the primary therapy for heart failure and atrial tachyarrhythmias until the discovery of mercurial and loop diuretics in the mid-20th century and β-blockers and calcium channel blockers in the late 20th century for the treatment of heart failure and atrial tachyarrhythmias, respectively [9]. Today, digoxin still has an important role in pharmacotherapeutics under the brand name Lanoxin^®^ although it is only applied under selective clinical situations [9,10]. However, a major clinical limitation of this class of compounds is their narrow therapeutic index, which is cause for concern in their wider applicability in the treatment of other diseases, including cancer [4].

Outside of their effects on the heart, cardenolides have been demonstrated to impact different mechanisms of proliferation in cancer cells. For example, ouabain, from *Strophanthus gratus* (Wall. and Hook.) Baill., suppressed the expression of STAT3, blocked STAT3-mediated transcription, and downstream target proteins independent of Na^+^/K^+^-ATPase activity [11]. Oleandrin from *Nerium oleander* L., ouabain, and digoxin all promote apoptosis in PC-3 prostate cancer cells by inducing the release of cytochrome c from the mitochondria and the proteolytic processing of caspase-8 and caspase-3 [12]. Further, oleandrin inhibited tumor necrosis factor (TNF)-induced activation of nuclear factor κB (NF-κB) and activator protein-1 (AP-1) in a concentration- and time-dependent manner [13].

Corchorusoside C (**1**), another cardenolide from *Streptocaulon juventas* (Lour.) Merr., has been demonstrated previously to inhibit the proliferation of the DU-145 prostate cancer cell line with a potent IC_50_ value of 80 nM [14]. Further, **1** was shown to have a significantly reduced cytotoxicity in the CCD-112CoN colon normal cells at an IC_50_ of 2.3 µM compared to HT-29 colon cancer cells at an IC_50_ value of 0.12 µM, resulting in a selectivity index of 22.5-fold toward cancerous colon cells [14]. Previous mechanistic studies revealed the ability of **1** to induce apoptosis in vitro by inhibiting the NF-κB inflammatory pathway and activate the intrinsic apoptotic pathway [14]. This was evidenced by a significant decrease in NF-κB p65 and p50, IKKα, and IKKβ protein expression levels and a significant increase in caspase-3, caspase-7, and PARP-1 expression levels following treatment with **1** in a concentration-dependent manner [14]. The NF-κB pathway is known to promote the survival, proliferation, and invasion of prostate cancer cells [15]. Thus, inhibition of this pathway by compound **1** could potentially be an effective treatment for prostate cancer cell proliferation, metastasis, or invasion of otherwise normal cells in the body. Further, the Poly (ADP-ribose) polymerase enzyme, PARP-1, is an integral protein in the DNA damage response mechanism ensuring the integrity of damaged DNA [16]. The overexpression of PARP-1, after treatment with **1** of DU-145 cells, suggests the effect that the cardenolide has on DNA damage and mitochondrial outer membrane permeabilization, as concluded in our previous study [14]. Protein expression levels of NF-κB and PARP-1-related proteins were corroborated by initial qualitative protein expression level studies in vivo using a zebrafish (*Danio rerio*) model [14]. Additionally, **1** was observed to induce less developmental toxicity after treatment of digoxin or cycloheximide for 24 h at the same dose of 50 µM in zebrafish 24 h post-fertilization (hpf) [14]. Thus, the favorable toxicity profile of **1** in vitro and in vivo and its activity against the DU-145 cell line supported its further study for its potential in the treatment of prostate cancer with fewer side effects and a greater therapeutic index than digoxin.

The present study aims to continue investigating the potential mechanism of action of corchorusoside C in a zebrafish model and in DU-145 prostate cancer cells to further evaluate its effects on the expression of PARP-1 and NF-κB pathway elements. In particular, those elements that provide insights into the selectivity of **1** towards the canonical or noncanonical NF-κB pathway. Additionally, since late-stage prostate cancer patients commonly experience pain in the hips, ribs, and spine due to tumor metastasis to these bones, further in vitro studies were performed to evaluate the effect of **1** on DU-145 cell migration in relation to prostate cancer cell metastasis [1]. Moreover, the production of the inflammatory cytokine IL-18 in the inflammasome has been shown to induce cell migration and invasion, so the effects of **1** on caspase-1 activity and IL-18 protein expression levels were examined in DU-145 cells [17]. Further, to evaluate the safety of **1**, this study aimed to determine the maximum toxicity dose (MTD) in zebrafish followed by a comparative toxicity study between digoxin and common anticancer molecules and **1**. Our findings suggest the significant activity of **1** on both the noncanonical NF-κB pathway and PARP-1 pathway elements as well as a higher tolerated dose of **1** in comparison to digoxin and other established anticancer drugs in the zebrafish model.

## 2. Results and Discussion

**Figure 1:**
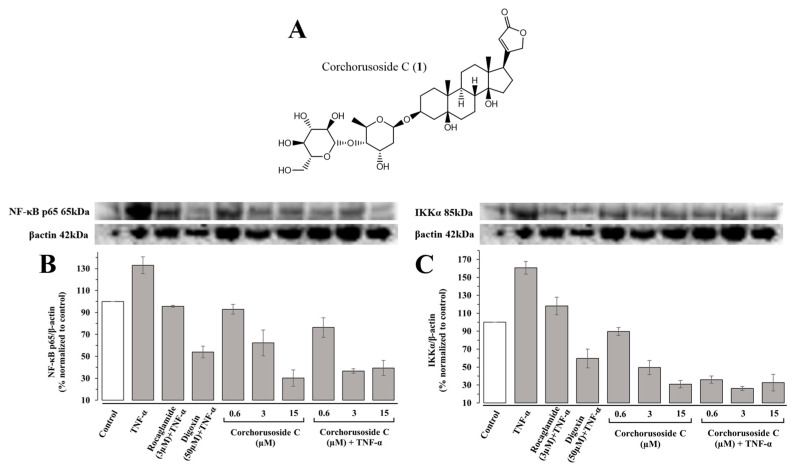
Effects of corchorusoside C (**1**) on NF-κB canonical and noncanonical pathway elements in zebrafish. (**A**) Structure of corchorusoside C isolated from *Streptocaulon juventas*. (**B**) Effects of **1** on the protein expression levels of NF-κB p65. (**C**) Effects of **1** on the protein expression levels of IKKα.

In previous mechanistic studies, corchorusoside C (**1**), shown in Figure 1A, exhibited significant effects on the protein expression levels of NF-κB and PARP-1 pathway elements in DU-145 prostate cancer cells and in zebrafish embryos [14]. This, along with its potent cytotoxic activity and selectivity for cancer cells, supports its further investigation into the selectivity of action over NF-κB or PARP-1 pathway elements [14].

The primary regulation of NF-κB is modulated by the IκB kinase (IKK) complex, which phosphorylates IκB (inhibitor of NF-κB) proteins [18]. The IKK complex is composed of IKKα and IKKβ subunits, which play important roles in noncanonical and canonical NF-κB signaling pathways, respectively, and the regulatory subunit IKKγ [19]. Activation of the IKKβ subunit in the IKK complex in the canonical pathway leads to the phosphorylation and polyubiquitination of IκBα [18,19]. This is the most well-studied protein of the IκB family, which is associated with the NF-κB p65/p50 heterodimer [18,19]. Subsequently, this results in the proteasomal degradation of IκB and translocation of the NF-κB p65/p50 heterodimer to the nucleus to activate target gene transcription [19]. If the noncanonical pathway IκB is not involved, the NF-κB-inducing kinase (NIK) activates IKKα, which then selectively phosphorylates NF-κB2 (p100) [18,19]. The p100 protein is a pro-form that is associated with RelB, a transactivation domain, and is proteolytically processed to p52 following its polyubiquitination [18,19]. The resultant p52-RelB heterodimer can activate the transcription of target genes [18,19]. Therefore, to investigate the role of **1** on the canonical and noncanonical NF-κB pathways, the protein expression levels of NF-κBp65 and IKKα were measured in immunoblot assays. First, TNF-α was introduced into a subset of zebrafish samples to induce NF-κB pathway activation and stimulation of the inflammatory pathway in otherwise healthy fish. Following treatment with **1**, zebrafish were euthanized via snap-freezing, homogenized, and used for immunoblotting assays, the results of which are shown in Figure 1B,C. Protein expression levels of both NF-κBp65 and IKKα were significantly downregulated in a concentration-dependent manner following treatment with **1** in both zebrafish subsets exposed to TNF-α and those that were not. In agreement with the previous study, the noncanonical NF-κB element, IKKα, was more affected by treatment with **1** than the canonical NF-κB element, NF-κB p65. For instance, a previous study demonstrated significant inhibitory activity of IKKα at lower doses than IKKβ in vitro, and the same effect was observed qualitatively in vivo [14].

**Figure 2:**
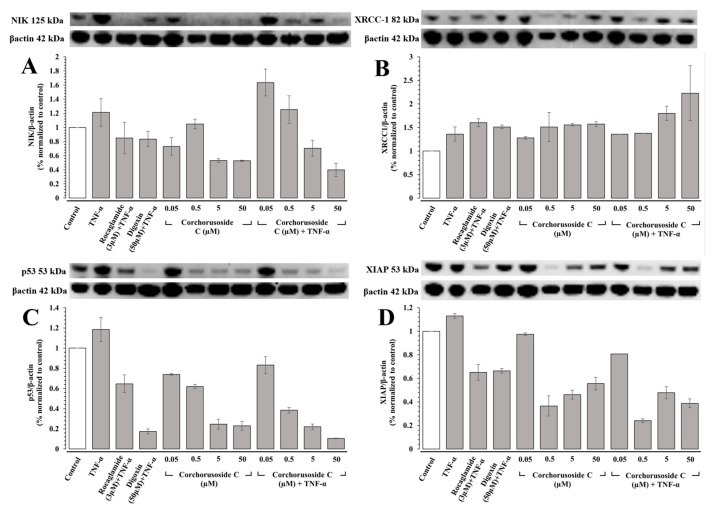
Effects of corchorusoside C (**1**) on NF-κB and PARP pathway elements. (**A**) Effects of **1** on the protein levels of NIK in DU-145 cancer cells. (**B**) Effects of **1** on the protein levels of XRCC-1 in DU-145 cancer cells. (**C**) Effects of **1** on the protein levels of p53 in DU-145 cancer cells. (**D**) Effects of **1** on the protein levels of XIAP in DU-145 cancer cells.

**Figure 3:**
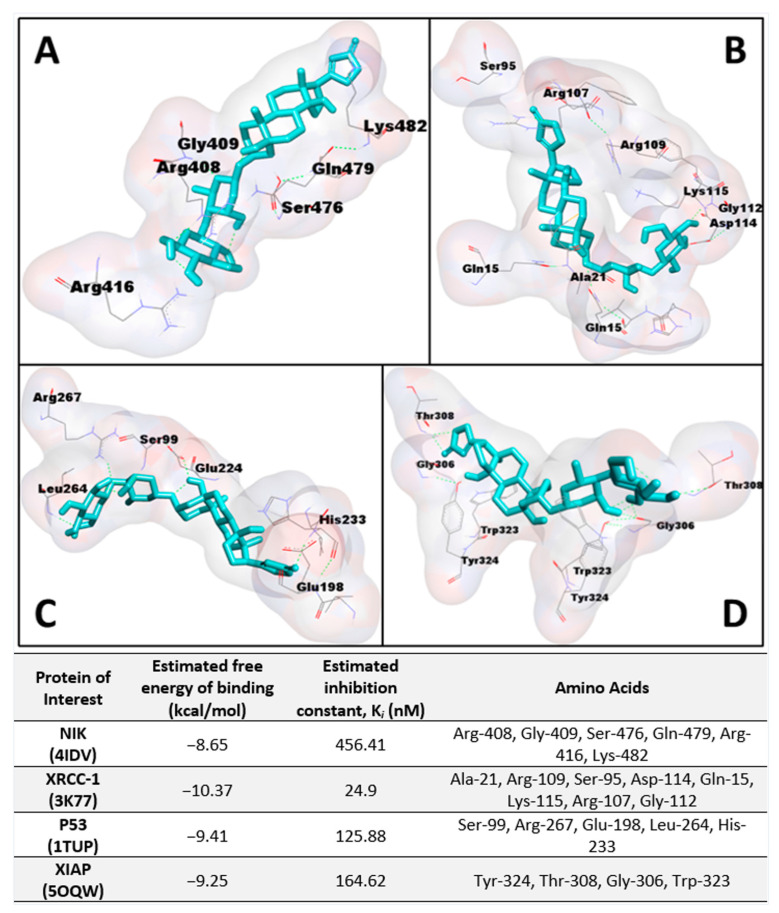
Molecular docking with various noncanonical NF-κB and PARP pathway elements. Docking of corchorusoside C (**1**, cyan) with PDB structures: (**A**) NIK (4IDV); (**B**) XRCC-1 (3K77); (**C**) P53 (1TUP); (**D**) XIAP (5OQW).

To confirm this activity on the noncanonical NF-κB pathway, protein expression levels of NIK were measured in DU-145 prostate cancer cells, as IKKα activity requires the activation of NIK [18]. Just as in the case of in vivo immunoblot studies in zebrafish, TNF-α was introduced into a subset of the cells to stimulate the NF-κB pathway in DU-145 cells. Protein expression levels of NIK, shown in Figure 2A, were downregulated significantly in both subsets of zebrafish, particularly in groups pretreated with TNF-α where a noticeable concentration-dependent response was observed. The affinity of **1** to NIK (PDB: 4IDV) was assessed in an in silico study to postulate a potential binding site and chain specificity. The results, shown in Figure 3A, demonstrated the binding of **1** to chain A of NIK with a potent K*_i_* value of 456.41 nM in a pocket formed by the amino acids Arg-408, Gly-409, Ser-476, Gln-479, Arg-416, and Lys-482.

PARP-1 is the founding member of the PARP family of proteins, containing a 50 amino-acid PARP binding motif signature in the C-terminal catalytic domain [20]. The enzymatic activity of PARP-1 is stimulated following DNA damage by interacting physically and functionally with proteins involved in the DNA repair pathways and recruiting proteins such as XRCC-1 to sites of DNA damage [20]. Corchorusoside C (**1**) was demonstrated to significantly increase the protein expression levels of PARP-1 in DU-145 cells at concentrations higher than 5 µM in a previous study [14]. Thus, to confirm that the PARP-1 activity is increased following treatment with **1**, protein expression levels of XRCC-1 were measured in DU-145 cells. The results, shown in Figure 2B, suggest that XRCC-1 activity is significantly increased following corchorusoside C treatment, further evidencing the activity of the cardenolide in inducing DNA damage. In silico docking study of **1** binding to XRCC-1 (PDB: 3K77) postulated the highest affinity of the ligand for chain C in a binding site composed of Ala-21, Arg-109, Ser-95, Asp-114, Gln-15, Lys-115, Arg-107, and Gly-112 with a K*_i_* value of 24.9 nM, as shown in Figure 3B. Current literature supports the functional cooperation between PARP-1 and p53, a tumor suppressor gene required for cell cycle checkpoints and apoptosis after cell death, in maintaining the genome [21]. A significant decrease in protein expression levels of p53 in a concentration-dependent manner was observed after treatment of **1** in DU-145 cells, as shown in Figure 2C. This is in agreement with a previous study of the cardiac glycosides, digoxin, and ouabain, reducing p53 levels in a time- and concentration-dependent manner in lung cancer cell lines [22]. Docking of **1** to p53 (PDB: 1TUP) revealed the highest binding affinity of **1** to chain B of the p53 protein in a binding pocket formed by Ser-99, Arg-267, Glu-198, Leu-264, and His-233 with a K*_i_* value of 125.88 nM shown in Figure 3C. Thus, the antiproliferative mechanism of **1** targets both the noncanonical NF-κB pathway and PARP-1 pathways following DNA damage. To investigate the selectivity of action for either of these pathways, the protein expression levels of X-chromosome-linked inhibitor of apoptosis protein (XIAP) were investigated. XIAP is an important antiapoptotic molecule that inhibits caspase-3 activation and the proteolytic cleavage of pro-caspase into an active form [23]. Caspase-3 activation leads to the cleavage and consequent inactivation of poly (ADP-ribose) polymerase (PARP-1), thereby allowing apoptosis to occur [21]. Further, XIAP expression has also been shown to positively be regulated by NF-κB and to protect endothelial cells from TNFα-induced apoptosis (Hida et al., 2000) [23]. TNF-α has also been demonstrated to induce PARP-1 activation in the absence of DNA damage, and TNFα-induced transcriptional activation of NF-κB requires PARP-1 activity [24]. Interestingly, in groups of DU-145 cells pretreated with TNF-α and not pretreated, XIAP expression was not significantly increased or decreased at the lowest dose used of 0.05 µM. However, XIAP expression was then significantly decreased at the next highest dose of 0.5 µM, above the reported IC_50_ value, in both the TNFα-treated and -untreated groups, followed by a steady dose-dependent increase in expression. These results, shown in Figure 2D, could be explained by the involvement of XIAP in both the NF-κB and PARP pathways. The dose-dependent increase in expression could be explained by the increased activity of XIAP in response to the increased expression of PARP-1 and caspase-3 proteins observed in the previous study after treatment with **1** at these higher doses [14]. The significant decrease in expression following the initial dose could be explained by the inhibition of NF-κB pathway elements at higher doses of **1**, which positively regulates XIAP expression [14,23]. A potential binding site for **1** on the XIAP protein (PDB: 5OQW) was investigated in molecular docking studies. The results, shown in Figure 3D, demonstrate the binding of **1** to chain B of XIAP with a K*_i_* value of 164.62 nM in a binding pocket formed by Tyr-324, Thr-308, Gly-306, and Trp-323.

**Figure 4:**
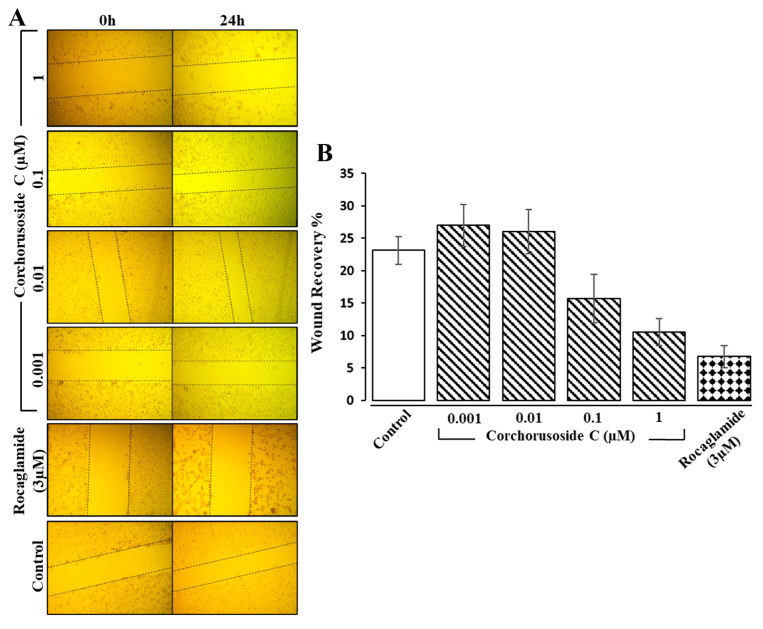
Effects of corchorusoside C (**1**) (0.001–1 μM) on the migration of DU-145 cells. (**A**) Representative histograms of the wound-healing assay displayed quantitatively as the percentage of wound recovery. (**B**) Representative images of the migration of DU-145 cells after treatment with **1**.

As mentioned previously, a common occurrence in late-stage prostate cancer is metastasis to the bones, particularly the hips, spine, and ribs; in cases of late-stage prostate cancer often causing pain and further complications in these patients [1]. Therefore, to analyze further the antiproliferative effects of **1** on DU-145 prostate cancer cell metastasis and invasion, an additional in vitro study was completed. A wound healing cell migration assay displayed evidence of a concentration-dependent suppression of DU-145 cell migration after 24 h of treatment with **1** (0.001–0.1 µM), as shown in Figure 4A,B. The results showed significant inhibitory effects of **1** on wound recovery at doses higher than 0.01 µM, suggesting that **1** might reduce the risk of prostate cancer metastasis and invasion. Digitoxin, a cardiac glycoside from foxglove, has also been shown to reduce the number of metastases in synergistic rats injected with PAIII rat prostate adenocarcinoma cells, further implicating this class of compounds’ ability to prevent prostate cancer metastasis and invasion [25]. Additionally, the inflammatory cytokine IL-18 has been demonstrated to be highly expressed in malignant tumors, including prostate cancer, and IL-18 production induces cell migration and invasion, increasing metastasis and tumor growth [17]. Therefore, an investigation into the protein expression levels of IL-18 after treatment with **1** and the activity of caspase-1 release of IL-18 in the inflammasome was investigated.

**Figure 5:**
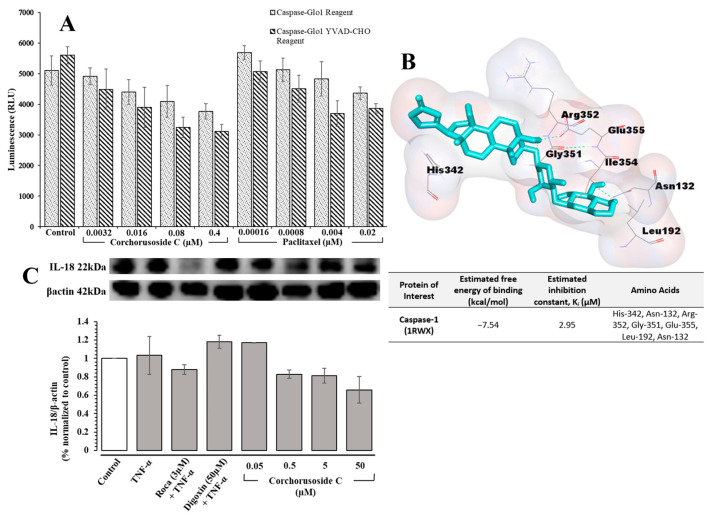
(**A**) Effects of **1** on caspase-1 activity in DU-145 cells using the Caspase-Glo^®^ 1 inflammasome assay from Promega. (**B**) Molecular docking with corchorusoside C (cyan) with caspase-1 (PDB structure: 1RWX) (**C**) Effects of **1** on the protein expression levels of IL-18 in DU-145 cancer cells.

The inflammasome is a protein complex that is formed in the cytoplasm in response to the binding of bacterial, viral, or other danger signals to NOD-like receptors (NLRs). Following binding, the pro-inflammatory caspase, caspase-1, is activated to convert the pro-inflammatory cytokines pro-IL-1β and pro-IL-18 into the inflammatory cytokines IL-1β and IL-18, respectively [26]. IL-1β and IL-18 release initiates the differentiation of inflammatory cells resultantly inducing an autoimmune response or the progression of tumors [26]. Therefore, the role **1** may play in the activation of inflammasome-associated pathways was investigated. The Caspase-Glo^®^ 1 assay from Promega was used to directly measure caspase-1 activity following corchorusoside C treatment of DU-145 prostate cancer cells. The results, shown in Figure 5A, demonstrate a significant dose-dependent reduction in caspase-1 activity after treatment with **1** compared to the control. This suggests that the apoptosis of DU-145 cells after treatment with **1** could also be due to the inhibition of caspase-1 activity in inflammasome activation. A molecular docking study of **1** for caspase-1 (PDB: 1RWX) suggested potent binding of this cardenolide to chain A of caspase-1 in a binding pocket formed by the amino acids His-342, Asn-132, Arg-352, Gly-351, Glu-355, Leu-192, and Asn-132 with a K*_i_* value of 2.95 µM, shown in Figure 5B. To confirm this activity, the protein expression levels of IL-18 after treatment with **1** were evaluated in DU-145 cells, as this inflammatory cytokine is released following caspase-1 activation [26]. As shown in Figure 5C, the expression levels of IL-18 were reduced significantly in a dose-dependent manner following treatment with **1,** further supporting the role of **1** in inhibiting inflammasome activation.

**Figure 6:**
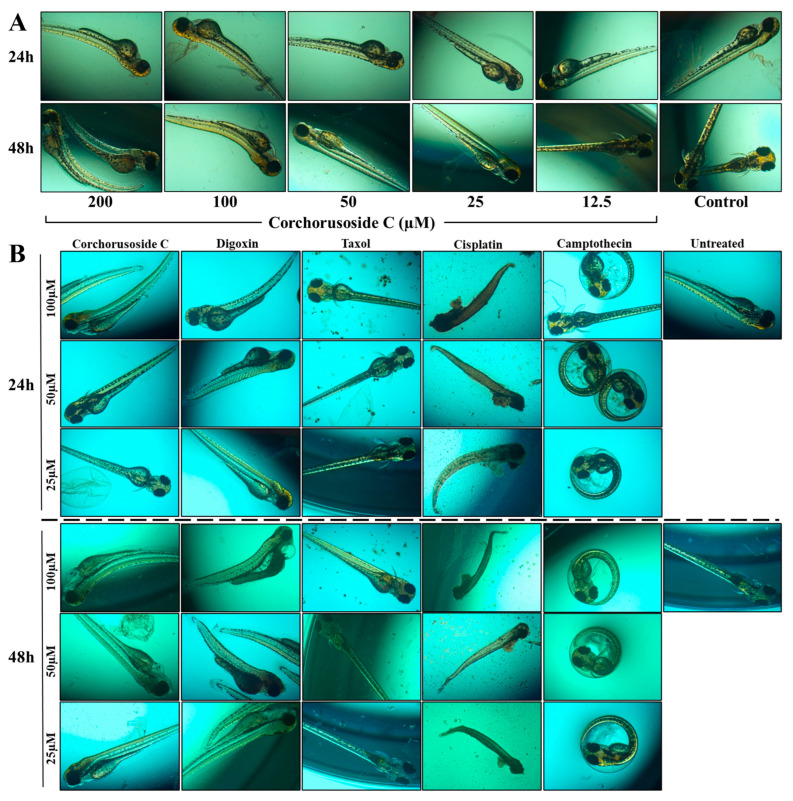
Corchorusoside C (**1**) toxicity profile. (**A**) Evaluation of the maximum toxicity dose (MTD) of corchorusoside C (0–200 μM) using a zebrafish model. (**B**) Zebrafish comparative toxicity study of current known anticancer molecules to corchorusoside C (25–100 μM) at 24- and 48-h of incubation with digoxin, paclitaxel, cisplatin, and camptothecin.

Due to the concern associated with the potential use of cardenolides in cancer treatment, zebrafish were also used to examine the maximum toxicity dose of **1** in vivo and, further, to develop a toxicity profile in relation to other common anticancer molecules. Corchorusoside C has been previously demonstrated to have no developmental toxicities at 50 µM, against zebrafish [14]. These results were replicated in a preliminary toxicity study in zebrafish at two different doses as two-fold dilution doses above and below this dose to evaluate the maximum tolerance of zebrafish to corchorusoside C. As displayed in Figure 6A, at doses higher than 50 µM visible abnormalities in zebrafish development became apparent as spine malformation or curvature was more frequent at these doses. These preliminary data suggested the maximum tolerated dose of corchorusoside C to be 50 µM, as at this dose no developmental abnormalities were observed. A further zebrafish study was completed to assess the safety of corchorusoside C in comparison to the current FDA-approved drugs digoxin, paclitaxel, and cisplatin, and the lead compound camptothecin. Toxicity was identified by observing phenotypic changes in zebrafish following treatment of each drug, all at one two-fold dose above and below the maximum tolerated dose of corchorusoside C determined in the preliminary study. Figure 6B represents the observed toxicities of at least in one fish after administration of each dose of the drug after 24- and 48-h incubation periods. Corchorusoside C demonstrated a more favorable toxicity profile at the maximum tolerated dose than any of the other drugs. After 24 and 48 h, zebrafish treated with 100 µM corchorusoside C were observed to have a spine malformation or curvature characteristic of the specific phenotype of an up-curved fish [27]. At the lower doses, this developmental abnormality was not prevalent even after 48 h. Treatment with the cardenolide digoxin resulted in an increase in the occurrence of the same spine malformation seen in corchorusoside C treatment at a lower dose of 50 µM after 24 h and 25 µM after 48 h, as shown in Figure 6B. Interestingly, an additional developmental abnormality characteristic of a short tail was seen in some of the fish at the 50 µM and 25 µM doses of the cardiac glycoside digoxin. After 24 h, the yolk sac of zebrafish treated with 50 µM and 100 µM of digoxin appeared to swell, and after 48 h it became necrosed and dark in color in comparison to the control [27]. Cisplatin treatment at all doses resulted in the complete death of all fish with noticeable phenotypic alterations such as a down-curved tail, complete destruction of the yolk sac, liver, heart, and stomach, and a short tail shown in Figure 6B [28]. Cisplatin is a first-line chemotherapeutic agent for prostate cancer, along with other platinum-based compounds that are associated with adverse side effects and nephrotoxicity [29]. As shown in Figure 6B, paclitaxel treatment at all concentrations produced an abundance of waste throughout the entire well, suggesting a potentially toxic effect on the zebrafish renal system. Nephrotoxicity is a common hazardous side effect in paclitaxel chemotherapy, as evidenced by its ability to induce apoptosis in mice kidney parenchymaous tissues in an in vitro study [30]. At all concentrations of camptothecin, an increase in the prevalence of zebrafish located within the chorion was observed, indicative of a general delay in development, as shown in Figure 6B [27]. Overall, at doses of 50 µM and 25 µM corchorusoside C demonstrated no toxicity to the developing zebrafish, suggesting a safer toxicity profile when compared at the same doses to digoxin, paclitaxel, cisplatin, and camptothecin after 48 h. The favorable toxicity profile of corchorusoside C compared to digoxin could be explained by their differences in structure, particularly in the saccharide moiety, as well as the position and stereochemistry of the hydroxy group substituents on their steroidal core [31]. Previous molecular docking studies of digoxin and its synthetic derivatives demonstrated the importance of the conformation of the hydroxy substituents of its steroid core, the lactone unit, and the saccharide moiety in cardenolides’ interaction with the Na^+^/K^+^-ATPase and other important targets of cancer [31]. Comparing the interactions of corchorusoside C and these same targets to their interactions with digoxin will be an important future objective in evaluating the ability of corchorusoside C to interact with important antiproliferative mechanisms, including NF-κB. This has been performed with another cardenolide, (+)-strebloside, which is structurally similar to corchorusoside C in the stereochemistry of the steroidal core and the lactone moiety but contains one less sugar unit in its saccharide component [32]. Compared to digoxin, (+)-strebloside seemed to be a more promising potential anticancer agent, as evidenced by the difference in their detailed direct binding interactions with the Na^+^/K^+^-ATPase and multiple components of the tumor microenvironment [32]. In particular, both were shown to bind directly with Kelch-like ECH-associated protein 1 (KEAP1), but only (+)-strebloside interacted with phosphoinositide 3-kinase (PI3K) [32]. Further, digoxin directly interacted with the NF-κB subunits p50, p52, and p65, while (+)-strebloside had limited interactions with these proteins [32]. Thus, the degree to which targets are modulated by these cardenolides is dependent on their structure. Corchorusoside C displayed a significant influence on noncanonical NF-κB elements in immunoblot studies and in our initial molecular docking studies. This, as well as its favorable toxicity profile in vivo, suggests the difference in binding interactions between corchorusoside C and digoxin in potential detailed molecular docking studies. Therefore, the results obtained from the initial molecular docking study, the in vitro and in vivo analyses on the NF-κB and PARP pathways, and the toxicity evaluations strongly encourage further studies on the potential of this cardenolide in the treatment of prostate cancer.

In conclusion, corchorusoside C (**1**) seems to act through the noncanonical NF-κB and PARP-1 pathways to induce apoptosis in the DU-145 prostate cancer cell line. Key elements of the NF-κB pathways, NF-κB p65, and IKKα were modulated by corchorusoside C in vitro, and these findings were confirmed in vivo in zebrafish. Other key proteins from the NF-κB noncanonical and PARP 1 pathways were also modulated in vitro in a concentration-dependent manner when treated with corchorusosisde C. In particular, protein expression of NIK in the noncanonical NF-κB pathway and XRCC-1 in the PARP-1 pathway. Furthermore, compared to common anticancer drugs and digoxin, corchorusoside C displayed an encouraging toxicity profile and was well tolerated at a maximum dose of 50 μM in zebrafish. Therefore, corchorusoside C has some potential in anticancer drug development and is worthy of future study for the treatment of prostate cancer.

## 3. Materials and Methods

### 3.1. Plant Material

The stems of *Streptocaulon juventas* (Lour.) Merr. were initially collected on a small scale in January 2010 from Turtle Conservation Site at Núi Chúa National Park, Vietnam, and a voucher specimen (AA06945/ST; voucher no. Soejarto et al. 14880) was deposited at the Field Museum of Natural History, Chicago, IL, USA, under accession number 2300834. A large-scale recollection of the same plant parts was made from the same location in July 2011 for isolation purposes and further studies.

### 3.2. Cell Culture

DU-145 cell lines were obtained from the American Type Culture Collection, Manassas, VA, USA). Cells were cultured in Dulbecco Modified Eagle Medium (DMEM) containing 10% fetal bovine serum and 1% antibiotic-antimycotic containing penicillin and streptomycin (Gibco, Rockville, MD, USA). The cells were grown in T75 tissue culture flasks as a monolayer and kept at 37 °C in an atmosphere with 5% CO_2_.

### 3.3. Western Blot Analysis

Zebrafish were housed in an automated fish housing system at 28.5 °C with a 14/10 h light/dark illumination scheme. At 72 h post-fertilization (hpf), zebrafish were treated with or without TNF-α for 1 h followed by treatment with corchorusoside C (**1**) (0.6–15 µM) for 3 h. Rocaglamide (3 µM) and digoxin (50 µM), both pretreated with TNF-α, were used as positive controls and normal unaltered fish water was used as the vehicle control. Then, the samples were euthanized via snap-freezing in dry ice and homogenized. For in vitro studies, DU-145 cells in DMEM medium were treated with or without TNF-α followed by treatment with **1** at different concentrations (0.05–50 µM) for 12 h. The BCA method was used to determine protein concentration for zebrafish and cell lysate samples and 20 µg of each sample was separated in Bolt 4–12% Bis-Tris Plus gels (Thermo Fisher Scientific, Waltham, MA, USA) by electrophoresis using SDS-PAGE. The proteins were transferred to a polyvinylidene fluoride membrane and blocked with 3% bovine serum albumin. Primary antibodies (NF-κB p65, IKKα, NIK, XRCC-1, p53, and XIAP), from Cell Signaling Technology, were incubated overnight followed by secondary antibody, from Santa Cruz Biotechnology, incubation for 1 h. The Supersignal Femto kit (Thermo Fisher Scientific, Waltham, MA, USA) was used to detect proteins and ImageJ 1.53e (NIH, Bethesda, MA, USA) was used to analyze band densities.

### 3.4. Molecular Docking

Molecular docking of corchrosusoside C (**1**) and the proteins NIK, XIAP, XRCC-1, p53, and caspase-1 using the crystallographic structures from the Protein Data bank website (PDB: 4IDV, 5OQW, 3K77, 1TUP, and 1RWX, respectively) was performed after removing ligands and heteroatoms. AutoDock Tools 1.5.6 (http://mgltools.scripps.edu/) was used to add polar hydrogens and assign Kollman charges of the edited protein structure. Compound **1** was inputted into AutoDock and optimized with each protein. All parameters were in their default setting in AutoDock apart from the Genetic Algorithm Parameters modified to 100 runs. The protein was held rigid while corchorusoside C was allowed to be flexible around the protein. The Grid box size was modified to be 126 Å × 126 Å × 126 Å in the x, y, and z dimensions with the center of the grid corresponding to the protein. The lowest estimated inhibition constant (K_i_) was used to determine the predicted docked protein-ligand complexes. The K_i_ value was calculated using the AutoDock program and the equation K_i_ = exp(ΔG × 1000/RT), where ΔG is the docking energy, R is the universal ideal gas constant (1.98719 cal K^−1^ mol^−1^), and T is the temperature (298.15 K).

### 3.5. Cell Migration Assay

A wound-healing assay was used to analyze the effect of corchorusoside C (**1**) on cell migration. DU-145 cells were seeded in a 24-well plate with 100% confluency. A scratch wound was created using a sterile 200 µL pipette tip. Cells were treated with corchorusoside C (0–1 μM) for 24 h. Images displaying wound recovery were taken with an Axiovert 40 CFL Zeiss microscope (magnifier Zeiss CP-Achromat 5×/0.12) and ProRes C10 plus camera. The ImageJ 1.53a program was used to calculate the wound recovery percent.

### 3.6. Caspase-1 Inflammasome Assay

Caspase-1 activity was evaluated using a modified protocol from Promega Caspase-Glo^®^ 1 inflammasome assay (G9951). DU-145 cells were grown in a white 96-well plate for 24 h to a density of 20 × 10^4^ cells/mL at 37 °C in a humidified 5% CO_2_ incubator. The cells were treated with **1** (0.0032–0.4 μM) or paclitaxel (0.00016–0.02 μM) for 24 h. Medium from each well (70 µL) was then discarded and the appropriate volume of Caspase-Glo 1 Reagent was added to half of the wells in the plate while Caspase-Glo 1 YVAD-CHO Reagent was added to the other half of the wells at a ratio of 1:1 sample volume to reagent volume. The plate was then incubated at room temperature and luminescence measured after 60 min.

### 3.7. Zebrafish Toxicity Assay

Zebrafish (*Danio rerio*) were provided by the Department of Neuroscience, the Ohio State University. Preliminary toxicity studies and comparative study experiments were completed under an approved animal protocol (Ohio State University IACUC protocol number 2014A00000006, entitled “Vertebrate Model for Natural Product Drug Discovery”; PI: Esperanza J. Carcache de Blanco). Fish were housed in an automated fish housing system at 28.5 °C. After 24 hpf zebrafish were exposed to corchorusoside C (12.5–200 μM) in a preliminary maximum toxicity dose study, followed by a comparative study of corchorusoside C with digoxin, paclitaxel, cisplatin, or camptothecin (25–100 μM). An untreated group of zebrafish in water was used as the vehicle control. The zebrafish were observed under an Axiovert 40 CFL Zeiss microscope (magnifier Zeiss CP-Achromat 5×/0.12) and pictures were taken using a ProRes C10 plus camera.

### 3.8. Statistical Analysis

Results obtained from experiments are presented as the means ± standard error of the mean (SEM). All measurements and analyses were carried out in triplicate in two or three independent experiments.

## Data Availability

Not applicable.
